# Synergistic contributions of periodic and aperiodic cortical activity to motor recovery prediction after stroke

**DOI:** 10.1093/braincomms/fcag400

**Published:** 2026-10-21

**Authors:** Seitaro Iwama, Daisuke Ito, Takayuki Kamimoto, Arisa Kawabata, Junsuke Ito, Tetsuya Tsuji, Junichi Ushiba, Michiyuki Kawakami

**Affiliations:** Department of Biosciences and Informatics, Faculty of Science and Technology, Keio University, Kanagawa 223-8522, Japan; Department of Rehabilitation Medicine, Keio University School of Medicine, Tokyo 160-8582, Japan; Department of Rehabilitation Medicine, Keio University School of Medicine, Tokyo 160-8582, Japan; Department of Rehabilitation Medicine, Keio University School of Medicine, Tokyo 160-8582, Japan; Department of Rehabilitation Medicine, Keio University School of Medicine, Tokyo 160-8582, Japan; Department of Rehabilitation Medicine, Keio University School of Medicine, Tokyo 160-8582, Japan; Department of Biosciences and Informatics, Faculty of Science and Technology, Keio University, Kanagawa 223-8522, Japan; Department of Rehabilitation Medicine, Keio University School of Medicine, Tokyo 160-8582, Japan

**Keywords:** prognosis prediction, sensorimotor rhythm, excitatory-inhibitory balance, neural plasticity, post-stroke hemiplegia

## Abstract

Functional motor recovery in chronic stroke can be promoted by intensive exercise aided by neurotechnology, yet reliable physiological markers to predict recovery remain limited. Scalp electroencephalography (EEG) is widely used in clinical routines, but conventional band-power metrics conflate oscillatory (periodic) and broadband (aperiodic) activity, limiting their interpretability. Here, we tested whether EEG spectral profiles predict motor recovery in chronic stroke. Resting-state EEG (1 min) was recorded at admission and discharge using a portable system (*N* = 41) and parameterized with a spectral decomposition model. Parameters were examined in relation to motor improvement, as indexed by gain in a clinical score, and used to construct a prediction model. Ipsilesional periodic activity at the individual alpha frequency (IAF) significantly increased after rehabilitation (*P* = 0.024) and correlated with motor improvement (*r* = 0.39, *P* = 0.010). A stepwise regression model identified higher IAF peaks, steeper aperiodic slopes, and their interaction as predictors of recovery (Severe group: *R*^2^ = 0.53, *P* = 0.003; balanced accuracy = 88%). The model outperformed a delta–alpha ratio–based approach commonly used in stroke prognosis. These findings indicate that the synergistic interaction between periodic and aperiodic activity provides a mechanistically meaningful and clinically relevant biomarker of residual plasticity, supporting individualized rehabilitation strategies in chronic stroke.

## Teaser

Iwama *et al*. report that 1-min resting electroencephalography recordings can help predict arm motor recovery after chronic stroke. Their findings show that the interaction between rhythmic and broadband cortical activity provides a clinically useful marker of recovery potential, supporting more individualized rehabilitation planning.

## Graphical Abstract

**Graphical Abstract:**
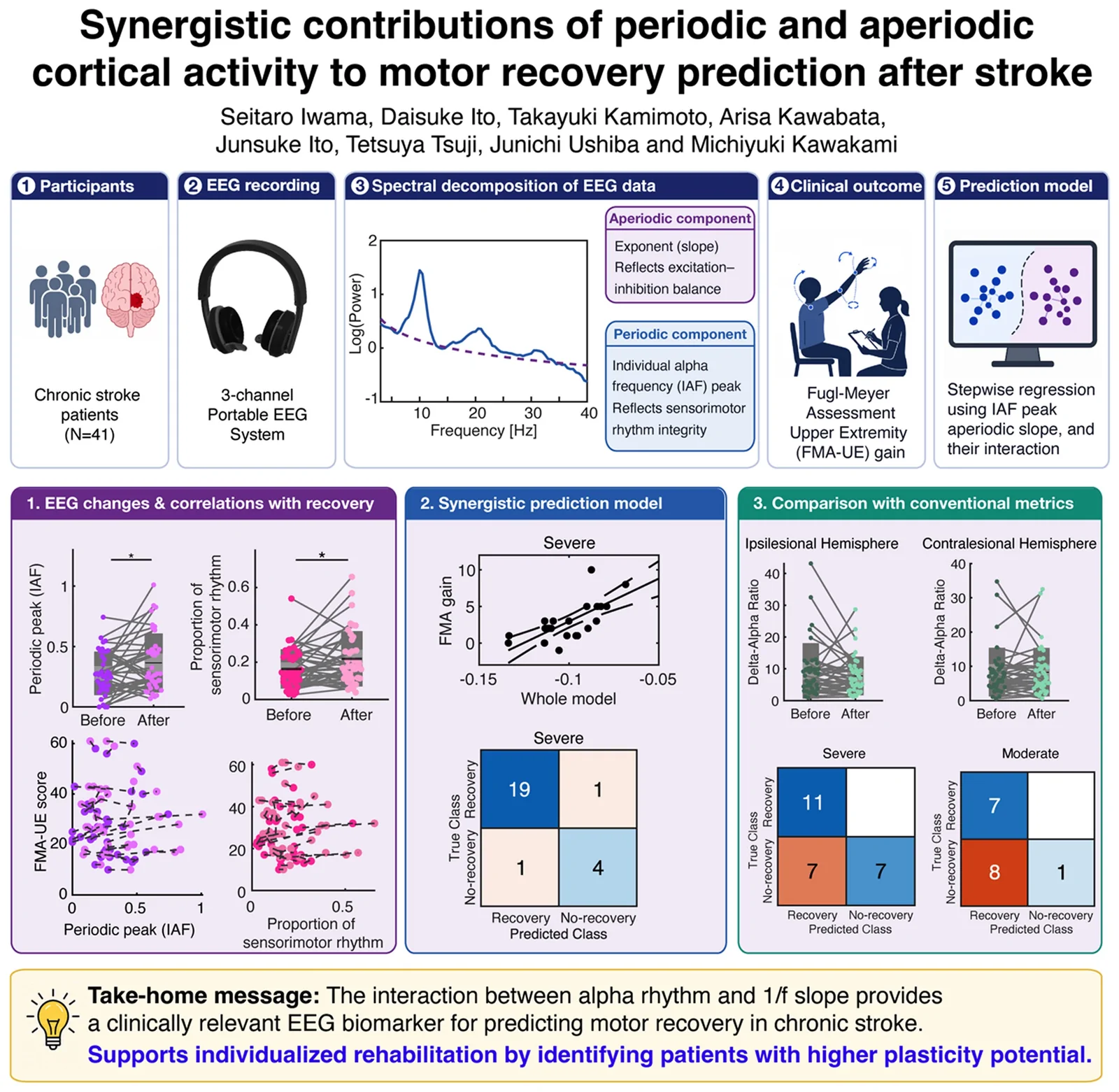

## Introduction

Motor recovery after stroke has been considered to plateau by the chronic stage, with most functional improvements believed to occur within the acute and early subacute periods.^1,2^ However, this consensus has been increasingly challenged by recent evidence demonstrating that meaningful gains can still be achieved through targeted and intensive interventions well beyond the early phase.^3,4^ Large-scale meta-analyses have supported the effectiveness of approaches such as robot-assisted therapy, neuromodulation, and high-dose task-specific training for chronic stage patients, especially when used in an individually-tailored manner.^5^ These findings highlight the persistent potential for neuroplasticity in chronic stroke and underscore the growing need for reliable physiological markers that can identify treatment-responsive patients and track intervention efficacy.

To evaluate and predict neurological recovery after stroke, cortical excitability and transcortical connectivity have been non-invasively captured through a multimodal framework, integrating structural information with functional magnetic resonance imaging^6,7^ (fMRI) or scalp electroencephalograms^8,9^ (EEG). In acute stroke, deviations from the proportional recovery rule, namely non-fitters,^10^ have been partially predicted by spectral features^11,12^ or connectivity metrics associated with population-level cortical dynamics.^8^ Although EEG-based metrics obtained in clinical practice have demonstrated relevance for patients in the acute to subacute phases, it remains unclear whether these metrics account for variability in recovery among patients in the chronic stage and how cortical spectral parameters are associated with recovery potential.

EEG-based neurophysiological characteristics are quantified using band-power metrics, which reflect the contributions of distinct neural populations. However, a key limitation is that they conflate oscillatory (periodic) and broadband (aperiodic) activity, making their physiological interpretation ambiguous.^13^ Periodic activity reflects narrowband neural oscillations generated by synchronized rhythmic firing of neuronal populations.^14^ Aperiodic activity, characterized by the 1/f-like spectral slope, reflects the broadband background neural activity and has been linked to cortical excitability and the overall balance of excitatory and inhibitory neural populations. In the stroke context, these decomposed metrics provide more mechanistically interpretable information than conventional band-power measures, as they can separately index oscillatory dynamics (e.g. sensorimotor rhythm integrity) and baseline cortical excitability state, both of which are disrupted after stroke. To address this, recent spectral analysis approaches have been developed that decompose EEG power spectra into periodic and aperiodic components, providing a more interpretable framework.^14^ The aperiodic component, quantified by parameters such as the aperiodic exponent (1/f-like spectral slope), is thought to reflect the excitation–inhibition balance of large-scale neural populations.^15,16^ In contrast, the periodic component, particularly sensorimotor activity expressed as the sensorimotor rhythm (SMR), has been widely studied,^17^ and resting-state SMR has been reported to correlate with better stroke recovery.^18^ However, the contribution of the aperiodic component, as well as the interaction between periodic and aperiodic activity, has not been systematically examined in relation to recovery.

In this study, we investigated whether EEG spectral profiles could predict motor recovery in chronic stroke patients. To test this, we recorded 1-min resting-state EEG using a portable system at admission and discharge and parameterized the power spectrum using a spectral decomposition model.^14^ These parameters were then used to train a statistical model aimed at predicting the degree of functional improvement, as measured by clinical scores. By identifying EEG-derived physiological markers that correlate with treatment responsiveness, the present study contributes to the development of objective, easily accessible tools for individualized rehabilitation planning in chronic stroke care.

## Materials and methods

### Study design

This study was conducted on patients who were admitted to the Department of Rehabilitation Medicine at Keio University Hospital between 24 April 2024, and 22 May 2025. The study protocol was approved by the institutional ethics review board at Keio University School of Medicine (Approval No. 2023-1210). Experiments were performed in accordance with the Declaration of Helsinki. All participants were inpatients for ∼3 weeks (25.0 ± 4 days) and received 1 h of standard occupational therapy combined with EMG-triggered functional electrical stimulation^19^ per day (5 days per week). The standard therapy included interventions such as range of motion training, task-oriented training, and training in activities of daily living. EMG-FES was applied to the affected upper extremity, targeting wrist and finger extensors, and was triggered by voluntary EMG activity detected from the paretic muscles. Resting-state EEG was measured for 1 min before and after the intervention period.

### Participants

A total of 50 post-stroke participants in the chronic stage (47 subcortical, 3 cortical) were enrolled in the study, all of whom completed the inpatient program. Of these, one was excluded due to inability to complete the pre-evaluation and eight were excluded due to insufficient EEG signal quality (high electrode impedance, *n* = 4; excessive body movement, *n* = 4), leaving 41 participants for analysis (see Supplementary Fig. 1 for a participant flow diagram). The mean age was 58.0 ± 9.9 years, with 19 males and 22 females (16 ischaemic, 25 haemorrhagic; Table 1). The time since stroke ranged from 5.9 to 252.6 months (mean: 72.4 ± 56.5 months); 1 participant was in the late subacute phase (3–6 months post-stroke) and 40 were in the chronic phase (>6 months post-stroke). The mean Fugl-Meyer Assessment for Upper Extremity^20^ (FMA-UE) score at the initial evaluation was 28.2 ± 12 (Fig. 1A).

**Figure 1:**
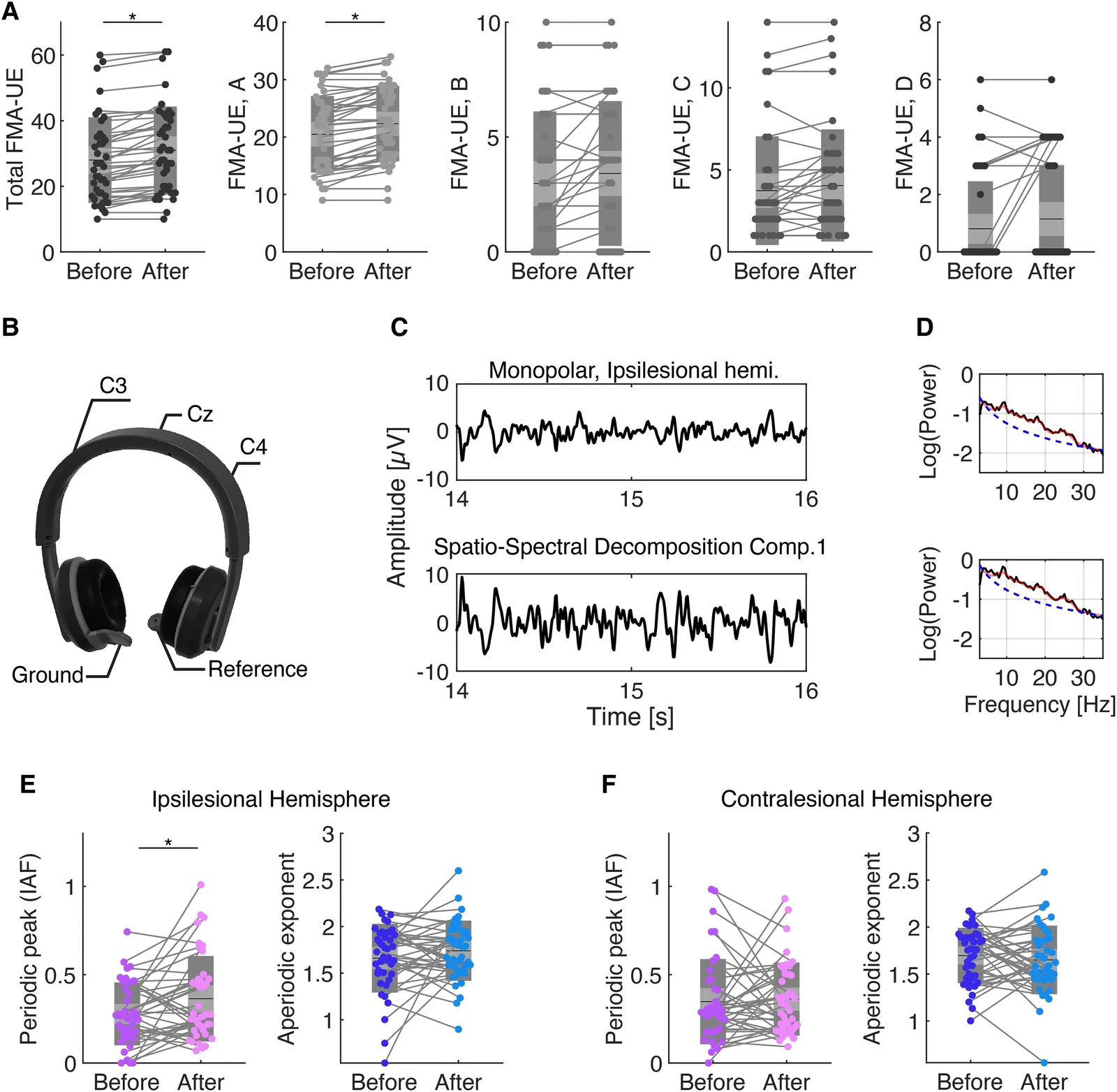
**Behavioural and electrophysiological changes following the intervention.** (**A**) Changes in Fugl-Meyer Assessment for Upper Extremity (FMA-UE) scores. Total scores and subscores are shown separately. (**B**) Schematic of the portable EEG setup. Three electrodes were positioned at C3, Cz, and C4, with the left ear and right cheek serving as the reference and ground, respectively. (**C**) Representative EEG signals from the ipsilesional hemisphere. The top panel shows raw monopolar recordings; the bottom panel shows the first component after preprocessing using spatio-spectral decomposition (SSD). (**D**) Power spectra of monopolar (top) and SSD (bottom) signals. Fits obtained with the *specparam* algorithm are shown, where the red line represents the full model and the blue line indicates the aperiodic component. (**E**) Changes in periodic and aperiodic EEG parameters in the ipsilesional hemisphere. The periodic peak at the individual alpha frequency and the aperiodic exponent are shown as pink and blue dots, respectively. Dark grey areas indicate ±1 standard deviation, light grey areas represent the 95% confidence interval, and the black line indicates the mean. A statistically significant increase in the periodic peak is marked with an asterisk (Paired *t*-test*, t*(40) = −2.34, *P* = 0.024, *d* = 0.37). (**F**) Changes in periodic and aperiodic EEG parameters in the contralesional hemisphere. Same format as in panel (**E**). Data points: in (**A**), each data point represents one participant (*N* = 41), with lines connecting the pre- and post-intervention values of the same participant; in (**E**) and (**F**), each data point represents one participant (*N* = 41) at the pre- or the post-intervention session; (**C**) and (**D**) show representative data from a single participant. Statistics: (**A**) Wilcoxon signed-rank test, Bonferroni-corrected for five comparisons (total score and subsections A–D); total FMA-UE, *W* = 16.5, *P* = 2.4 × 10^−7^ (corrected *P* = 1.2 × 10^−6^); subsection A, *W* = 13.0, *P* = 5.8 × 10^−7^ (corrected *P* = 2.9 × 10^−6^); subsection B, *W* = 8.0, *P* = 0.013 (corrected *P* = 0.064); subsection C, *W* = 40.0, *P* = 0.071 (corrected *P* = 0.36); subsection D, *W* = 7.0, *P* = 0.057 (corrected *P* = 0.29). (**E**) Paired *t*-test; periodic peak at IAF, *t*(40) = −2.34, *P* = 0.024, Cohen’s *d* = 0.37; aperiodic exponent, *t*(40) = −1.28, *P* = 0.21, *d* = 0.20. (**F**) Paired *t*-test; periodic peak at IAF, *t*(40) = −0.34, *P* = 0.74, *d* = 0.05; aperiodic exponent, *t*(40) = 0.78, *P* = 0.44, *d* = 0.12.

**Table 1:** Patient demographics

	Mean	SD
Age, years	58.0	9.92
Female	22 (53.7%)	
Time since stroke, months	72.4	56.5
Length of hospital stay	24.95	4.06

	Count
Lesion side, Left/Right	23/18
Infarction/Haemorrhage	16/25
Damage site	39 subcortical/2 cortical

	Median	IQR
Stroke Impairment Assessment Set (SIAS) proximal	3	1
SIAS extremity	2.5	2
SIAS sensory touch	2	0
SIAS sensory position	2	2

	Mean	SD
Fugl-Meyer Assessment Score	28.2	12.8

### Signal acquisition

EEG signals were acquired using an amplifier (AE-120A, Nihon Kohden) combined with a custom-made electrode holder (Fig. 1B). The signals were recorded monopolarly from C3, C4, and Cz according to the international 10–20 system, with the reference electrode placed behind the ear and the ground electrode on the cheek. The sampling rate was 200 Hz. The impedance for all three electrodes was maintained below 50 kΩ. Before the 1-min eyes-open resting-state EEG measurement, participants were instructed to remain as still as possible, refrain from blinking, and avoid concentrating on any specific thought. The power spectrum of the acquired EEG signals was calculated on-site for quality checking. EEG data were visually inspected by a trained operator, and segments containing excessive muscle artefacts, eye movement artefacts, or electrode drift were identified. Participants were excluded if more than 50% of the 1-min recording was contaminated by artefacts or if the signal quality was judged insufficient for reliable spectral estimation.

### Signal processing

The EEG signals were re-referenced to the lesioned hemisphere and Cz, bandpass-filtered (0.5–40 Hz), and transformed into power spectra using Welch’s method (1-s Hanning window, 50% overlap; power values reported in log10 (µV^2^/Hz) scale). Spectra were parameterized with the specparam^14^ algorithm (frequency range: 1–40 Hz; fixed aperiodic mode without knee; peak width limits: 1–8 Hz; maximum number of peaks: 6; minimum peak height: 0.1; peak threshold: 2.0 standard deviations, Supplementary Figs 2 and 3) to identify the individual alpha frequency (IAF). Rhythmic features at the IAF were then extracted using spatio–spectral decomposition^21^ (SSD), and the components with maximal weights over the lesioned and non-lesioned hemispheres were retained for analysis (Fig. 1C).

SSD-derived signals were analyzed in two ways: (1) spectral features, including the delta–alpha ratio (DAR, 0.5–4 Hz/8–13 Hz), computed from specparam-parameterized spectra, that is the full model fit of *specparam* algorithm; and (2) temporal dynamics, quantified with the fBOSC algorithm,^22^ which detects sustained high-amplitude oscillations exceeding amplitude (set by the aperiodic fit) and duration (≥4 cycles) thresholds. The latter was used to calculate the occurrence rate of high-amplitude oscillations at the IAF.

### Prognosis prediction model for functional motor recovery

We constructed a model to estimate the FMA gain from features obtained from the pre-intervention EEG. The features used were those extracted from signals originating from the lesioned and non-lesioned hemispheres, specifically IAF peak and aperiodic exponent from Specparam, and the burst duration from fBOSC. A regression model was built using stepwise regression. The performance of this model in predicting whether the prognosis would improve or not was evaluated by calculating the balanced accuracy in a leave-one-out cross-validation (LOCCV) given by Equation (1).


(1) $$\mathrm{Balanced}\;\mathrm{Accuracy}\;=\frac{\mathrm{TPR}+\mathrm{TNR}}{2}$$

The accuracy was also evaluated using the DAR in the same manner. To account for differences in biological characteristics, the analysis was performed separately for Severe (FMA-UE score < 30) and Moderate (FMA-UE score ≥ 30) groups.^23^

To evaluate whether the regression models were driven by a small number of influential observations, we performed post-hoc influence diagnostics. For each subgroup model, we computed Cook's distance, leverage (h_ii), and studentized residuals. Observations exceeding Cook's distance > 4/n or leverage > 2p/n were flagged as potentially influential. We then (i) re-estimated the model after excluding flagged observations and compared coefficient estimates, standard errors, *R*^2^, and overall *P*-values, and (ii) performed a leave-one-out re-estimation to quantify the stability of each coefficient (mean, SD, min, max across LOO iterations).

### Statistical analysis

For paired data, we used a paired *t*-test for parametric data and the Wilcoxon signed-rank test for non-parametric data. When performing correlation analysis on multiple samples from a single participant, repeated measures correlation analysis^24^ was used. Normality of EEG power-related variables was assessed using the Shapiro–Wilk test; where normality was violated, non-parametric alternatives (Wilcoxon signed-rank test) were applied. The false positive rate for multiple comparisons was corrected with the Bonferroni method. For the predictive model, the primary performance was evaluated by leave-one-out cross-validation (LOOCV) using root-mean-squared error (RMSE), mean absolute error (MAE), and out-of-sample coefficient of determination (*R*^2^) between predicted and observed FMA gain; 95% confidence intervals were obtained by 1000-pair bootstrap, and statistical significance was assessed by a 2000-iteration label-permutation null that re-ran the full LOOCV pipeline on shuffled outcomes. A secondary classification analysis defined ‘improvement’ a priori as ΔFMA-UE ≥ 5 points (the minimal clinically important difference for the upper-extremity Fugl-Meyer^25^).

## Results

### Post-stroke motor recovery in the chronic stage

The patients who underwent intensive rehabilitation exhibited systematic improvement in motor ability. The FMA-UE scores were measured before and after the intervention (Fig. 1A).

We found a significant increase in the total FMA score and the subsection for shoulder movement (i.e. subsection A, Wilcoxon signed-rank test, *P* < 0.05 with Bonferroni correction). The total FMA-UE score increased significantly (Wilcoxon signed-rank test, *W* = 16.5, *P* = 2.4 × 10^−7^, Bonferroni-corrected *P* = 1.2 × 10^−6^), as did subsection A (*W* = 13.0, *P* = 5.8 × 10^−7^, corrected *P* = 2.9 × 10^−6^), whereas subsections B–D did not (B: *W* = 8.0, *P* = 0.013, corrected *P* = 0.064; C: *W* = 40.0, *P* = 0.071, corrected *P* = 0.36; D: *W* = 7.0, *P* = 0.057, corrected *P* = 0.29; correction over five comparisons).

The patients showed 2.95 ± 2.9 and 1.85 ± 1.6 improvement, respectively. Given that minimal clinically important difference for FMA-UE is around 4.25–7.25,^25^ 10 out of the 41 patients showed meaningful recovery after rehabilitation.

### Ipsilesional neural oscillations were modulated after rehabilitation

We next sought the neurophysiological changes that accompanied the functional motor recovery. To this end, the resting EEG signals were analyzed using Welch’s method, and their periodic and aperiodic components were parameterized (Fig. 1D). The parameters were compared between the two time points (Fig. 1E and F). We found a significant increase in the spectral power peak at IAF derived from the ipsilesional hemisphere (Paired *t*-test, *t*(40) = −2.34, *P* = 0.024, *d* = 0.37) while aperiodic components (*t*(40) = −1.28, *P* = 0.21) and parameters derived from the contralesional hemisphere did not (Periodic peak: *t*(40) = −0.34, *P* = 0.74; Aperiodic exponent: *t*(40) = 0.78, *P* = 0.44). Effect sizes for the remaining comparisons were small (ipsilesional aperiodic exponent, *d* = 0.20; contralesional periodic peak at IAF, *d* = 0.05; contralesional aperiodic exponent, *d* = 0.12).

Using the two neural features, we next tested if FMA scores were associated with these neural features (Fig. 2A and B). We examined the repeated-measures correlation with the FMA score and each feature derived from the bilateral hemispheres. A significant positive correlation was found between the FMA score and the ipsilesional periodic peak (repeated measures correlation, $r_{m}$ = 0.38, *P* = 0.014), indicating that increased oscillatory power at IAF is associated with better motor function. The remaining associations were not significant (ipsilesional aperiodic exponent, *r*_rm_ = 0.23, *P* = 0.15; contralesional periodic peak at IAF, *r*_rm_ = 0.07, *P* = 0.67; contralesional aperiodic exponent, *r*_rm_ = −0.12, *P* = 0.46; df = 40 for all comparisons).

**Figure 2:**
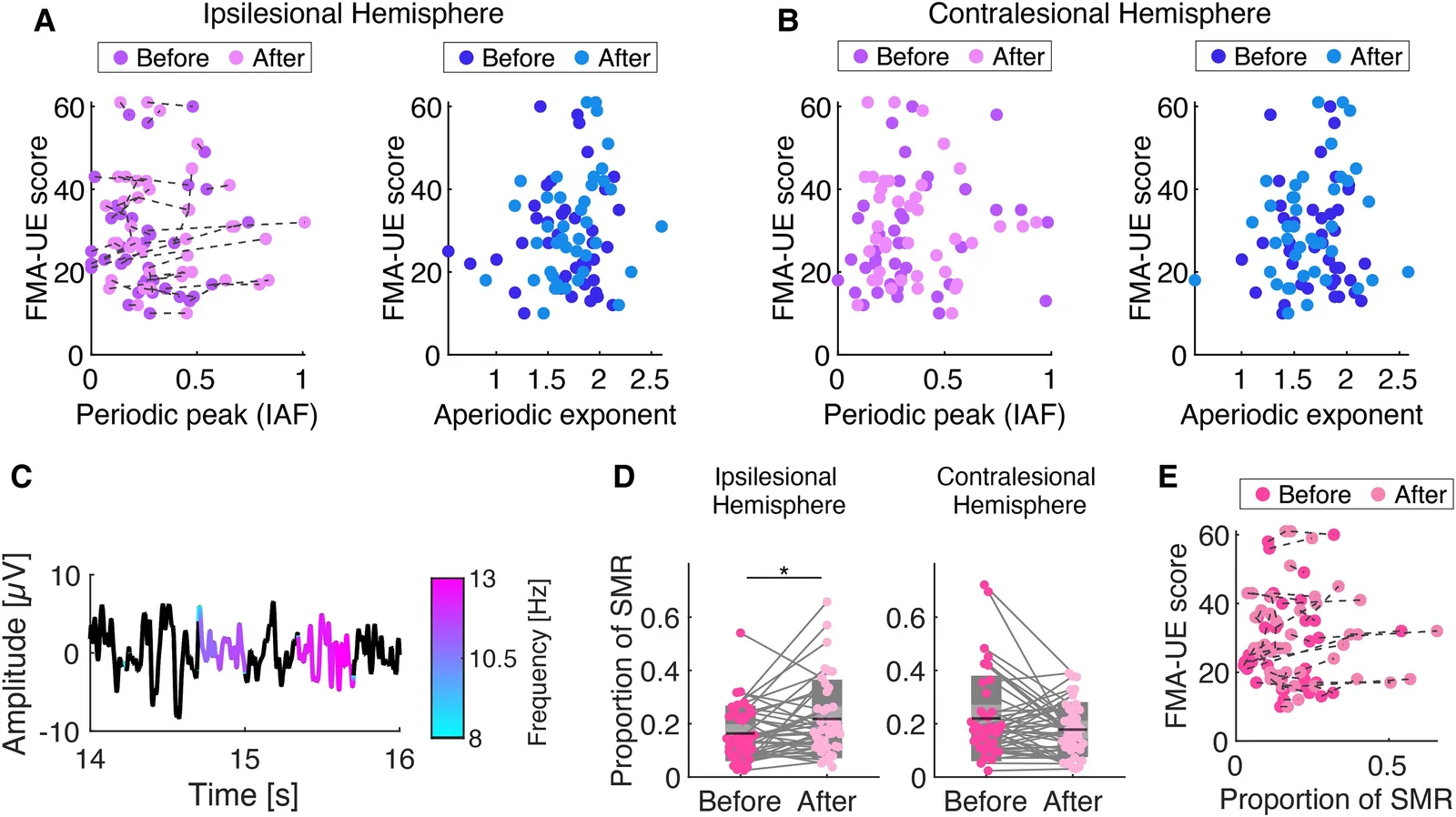
**Relationship between FMA-UE scores and EEG power spectral parameters.** (**A**) Scatter plots showing the relationship between FMA-UE scores and periodic/aperiodic power spectral density (PSD) parameters from the ipsilesional hemisphere. The left panel displays periodic peak values (individual alpha frequency), and the right panel shows aperiodic exponents. (**B**) Scatter plots of FMA-UE scores and PSD parameters from the contralesional hemisphere. The layout is the same as in (**A**). (**C**) Representative example of high-amplitude signals detected using an oscillation detection algorithm. Only alpha oscillations are highlighted with colours corresponding to their frequencies. High-amplitude signals at lower frequencies (e.g. around 14.5 s) remained undetected. The bottom panel indicates high-amplitude time-series at 12.5 Hz with a sinusoidal example. (**D**) Proportion of sensorimotor rhythm at IAF before and after the intervention. The left and right panels represent data from the ipsilesional and contralesional hemispheres, respectively. A significant increase was observed only in the ipsilesional hemisphere (paired *t*-test, *t*(40) = −2.52, *P* = 0.016, *d* = 0.39). (**E**) Scatter plot showing the relationship between FMA-UE scores and the proportion of SMR derived from the ipsilesional hemisphere (repeated measures correlation, $r_{m}$ = 0.39, *P* = 0.010). Data points: in (**A**), (**B**) and (**E**), each data point represents one measurement session of one participant (41 participants × 2 sessions = 82 data points), and the line shows the within-participant (repeated measures) fit; in (**D**), each data point represents one participant (*N* = 41) at the pre- or the post-intervention session; (**C**) shows representative data from a single participant. Statistics: (**A**) Repeated measures correlation (df = 40); periodic peak at IAF, *r*_rm_ = 0.38, *P* = 0.014; aperiodic exponent, *r*_rm_ = 0.23, *P* = 0.15. (**B**) Repeated measures correlation (df = 40); periodic peak at IAF, *r*_rm_ = 0.07, *P* = 0.67; aperiodic exponent, *r*_rm_ = −0.12, *P* = 0.46. (**D**) Paired *t*-test; ipsilesional hemisphere, *t*(40) = −2.52, *P* = 0.016, Cohen’s *d* = 0.39; contralesional hemisphere, *t*(40) = 1.73, *P* = 0.091, *d* = 0.27. (**E**) Repeated measures correlation, *r*_rm_ = 0.39, *P* = 0.010, df = 40.

In addition to the static averaged spectral features, we investigated the temporal dynamics of the alpha oscillations. Specifically, we investigated sustained high-amplitude oscillation at IAF, which reflects the SMR related to the resting-state cortical idling state in the sensorimotor cortex^26^ (Fig. 2C). The comparison at pre- and post-evaluation revealed that significant increase in the burst probability at IAF in the ipsilesional hemisphere (Fig. 2D, Paired *t*-test, *t*(40) = −2.52, *P* = 0.016, *d* = 0.39) but not in the contralesional hemisphere (Paired *t*-test, *t*(40) = 1.73, *P* = 0.09, *d* = 0.27). Further, the proportion of SMR at IAF, derived from the ipsilesional hemisphere, was significantly correlated with the total FMA-UE score (Fig. 2E, Repeated measures correlation, $r_{m}$ = 0.39, *P* = 0.010). Collectively, the oscillatory dynamics on the ipsilesional side are associated with functional motor recovery in the chronic stage.

### Prognosis model for functional recovery based on neurophysiological characteristics at the pre-intervention period

Having established that resting-state EEG characteristics were related to arm motor function after stroke, we then investigated whether a combination of neural features measured before the intervention contributes to the prediction of outcome changes (Fig. 3). We constructed a stepwise regression model that used periodic and aperiodic neural features derived by the specparam and fBOSC algorithms.

**Figure 3:**
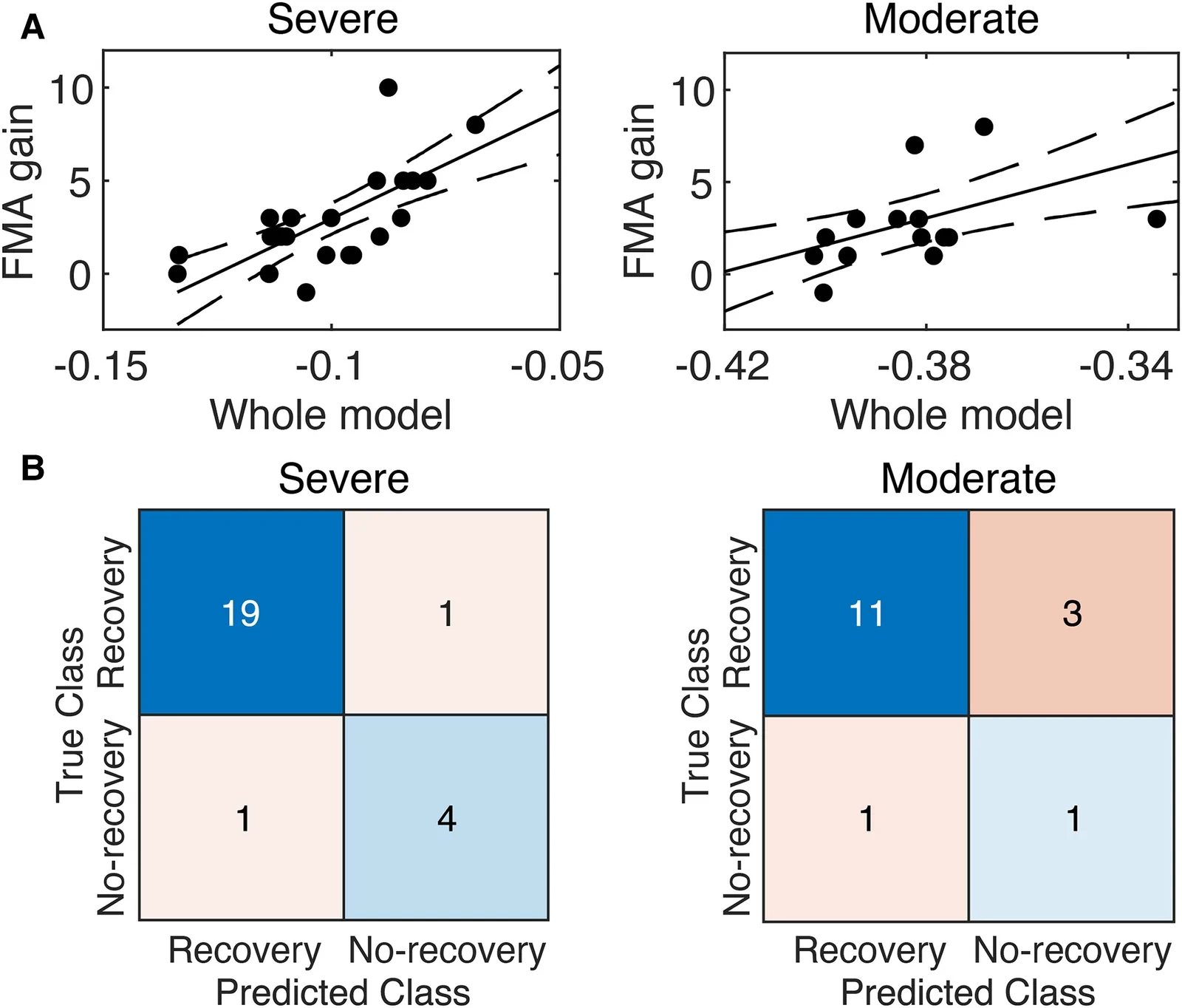
**Prognostic prediction model using the interaction of periodic and aperiodic EEG components.** (**A**) Results of the stepwise regression model for patients with severe and moderate impairments. The regression model was constructed using the full dataset. (**B**) Confusion matrices for recovery prediction using a leave-one-out cross-validation procedure. Results are shown separately for patients with severe and moderate impairments. Data points: in (**A**), each symbol is a regression coefficient (± standard error) of the stepwise model, estimated from one observation per participant (Severe, *n* = 25; Moderate, *n* = 16); in (**B**), each participant contributes one left-out prediction to the confusion matrix (severe, *n* = 25; Moderate, *n* = 16). Statistics: (**A**) Stepwise multiple linear regression; coefficient *P* values are two-sided *t*-tests. Severe group: *R*^2^ = 0.53, *F*(4,20) = 5.67, *P* = 0.003; ipsilesional periodic peak at IAF, *β* = −45.0, *P* = 0.0078; ipsilesional aperiodic exponent, *β* = −10.1, *P* = 0.011; ipsilesional SMR proportion, *β* = −29.8, *P* = 0.0018; periodic × aperiodic interaction, *β* = 38.8, *P* = 0.0017. Moderate group: *R*^2^ = 0.50, *F*(3,12) = 4.02, *P* = 0.034; ipsilesional periodic peak at IAF, *β* = −58.9, *P* = 0.0048; ipsilesional aperiodic exponent, *β* = −18.1, *P* = 0.0088; periodic × aperiodic interaction, *β* = 38.4, *P* = 0.0062. (**B**) Leave-one-out cross-validation; the matrices are shown at the per-fold optimal cut-off (balanced accuracy = 0.88 for the Severe group and 0.64 for the Moderate group). Continuous accuracy for the Severe group: RMSE = 2.56 (95% CI 1.77–3.30), MAE = 2.06, out-of-sample *R*^2^ = 0.20, Pearson *r* = 0.55 between predicted and observed FMA-UE gain (label-permutation *P* = 0.004, 2000 iterations; Spearman *ρ* = 0.53, *P* = 0.007); Moderate group: RMSE = 3.77, MAE = 2.71, out-of-sample *R*^2^ = −0.65, Pearson *r* = 0.01, permutation *P* = 0.30. At the pre-specified MCID cut-off (ΔFMA-UE ≥ 5 points), balanced accuracy was 0.786 for the Severe group (sensitivity = 0.57, specificity = 1.00, positive predictive value = 1.00, Matthews correlation coefficient = +0.70) and 0.462 for the Moderate group.

We sought the neural features that contributed to the prediction with the participants stratified as severe according to the FMA-UE score. The full model constructed using all participants labelled as severe showed a statistically significant model explaining FMA-UE gain (Fig. 3A, Full model: *P* = 0.003, *F*(4,20) = 5.67, $R^{2}=\;0.53$). This model was composed of the following five terms: intercept, ipsilesional periodic peak at IAF (*β* = −45.0, *P* = 0.0078), ipsilesional aperiodic exponent (*β* = −10.1, *P* = 0.011), ipsilesional SMR proportion (*β* = −29.8, *P* = 0.0018), and the interaction between periodic peak and aperiodic exponent (*β* = 38.8, *P* = 0.0017).

For the patients stratified as moderate, the stepwise model (Full model: *P* = 0.034, *F*(3,12) = 4.02, $R^{2}=\;0.50$) was comprised of the following three terms: ipsilesional periodic peak at IAF (*β* = −58.9, *P* = 0.0048), ipsilesional aperiodic exponent (*β* = −18.1, *P* = 0.0088), and their interaction (*β* = 38.4, *P* = 0.0062).

Next, we evaluated the predictive accuracy of the models using a LOOCV procedure (Fig. 3B). The primary evaluation was continuous. For the Severe group, LOOCV yielded RMSE = 2.56 (95% CI 1.77–3.30), MAE = 2.06, and out-of-sample *R*^2^ = 0.20, with a significant correlation between predicted and observed FMA gain (Pearson *r* = 0.55, permutation *P* = 0.004, 2000 iterations; Spearman *ρ* = 0.53, *P* = 0.007). For the Moderate group, LOOCV yielded RMSE = 3.77, MAE = 2.71, and out-of-sample *R*^2^ = −0.65 (Pearson *r* = 0.01, permutation *P* = 0.30), indicating that the Moderate-group model did not generalize out of sample. For each iteration, a model was constructed using the dataset excluding one patient, and the model was then used to predict the outcome for the left-out patient. The confusion matrix at the optimal cut-off value at each iteration (note that the cut-off value was determined based on the training dataset to avoid leakage) was shown. The ‘improvement’ label was defined based on the MCID threshold (FMA-UE gain ≥ 5 points). The balanced accuracy for the severe patients was 0.88 and that for moderate patients was 0.64. At the pre-specified MCID cut-off (ΔFMA-UE ≥ 5 points), balanced accuracy was 0.786 for Severe (sensitivity = 0.57, specificity = 1.00, positive predictive value (PPV) = 1.00, Matthews correlation coefficient (MCC) = +0.70; 4 of 7 MCID-level improvers correctly detected with zero false alarms) and 0.462 for Moderate; the per-fold ‘optimal’ cut-off was stable in the Severe stratum (median 3.22, range 3.05–5.13) but highly unstable in the Moderate stratum (range −1.00–8.00), and we therefore retain the classification analysis as clinically deployable only for the Severe group. In the Severe group, the interaction between periodic peak and aperiodic exponent was retained in 23 of 25 LOOCV folds, demonstrating reasonable feature selection stability. Models constructed in each iteration also contained terms found in the full dataset.

In the Severe group (*n* = 25, Supplementary Fig. 4), 1 observation exceeded the Cook's distance threshold of 4/*n* = 0.160 (max Cook's D = 1.09; max leverage = 0.70). After excluding this observation, the model retained a similar structure: *R*^2^ changed from 0.532 to 0.576 (adj. *R*^2^: 0.438 → 0.487), overall *P* from 0.0032 to 0.0019, and the sign of the interaction of periodic and aperiodic interaction term was preserved (*β*_full = 38.84, *β*_trimmed = 57.46). Leave-one-out re-estimation yielded coefficient SDs of ≤ 6.16 for all retained terms, and no single observation removal shifted the overall model *P*-value above 0.05 (LOO pmax = 0.035). A parallel analysis in the Moderate group (*n* = 16) is reported in Supplementary Fig. 5. These results indicate that the reported associations in the severe group are not driven by a few isolated participants with unusually large recovery. In contrast, the Moderate-group model was sensitive to a small number of influential observations (max Cook's *D* = 3.57); following their exclusion, model significance was not retained (*P* = 0.308).

### Conventional frequency band ratio measures are associated with baseline motor function

Finally, we compared the prediction accuracy of the proposed model with a previously reported band-power metrics, namely DAR (Fig. 4). Since DAR is calculated based on the power spectrum without spectral detrending, we first tested if DAR correlated with the periodic and aperiodic component. As expected, DAR was significantly correlated with periodic peaks at IAF and aperiodic exponents (Fig. 4A, Repeated measures correlation, ipsilesional hemisphere: periodic peak at IAF, $r_{m}$ = −0.375, *P* = 0.014, aperiodic exponent, $r_{m}$ = 0.367, *P* = 0.016; contralesional hemisphere: periodic peak at IAF, $r_{m}$ = −0.52, *P* < 0.001, aperiodic exponent, $r_{m}$ = 0.16, *P* = 0.31), indicating that the frequency band ratio is determined by both the contribution of periodic and aperiodic components in the power spectra.

**Figure 4:**
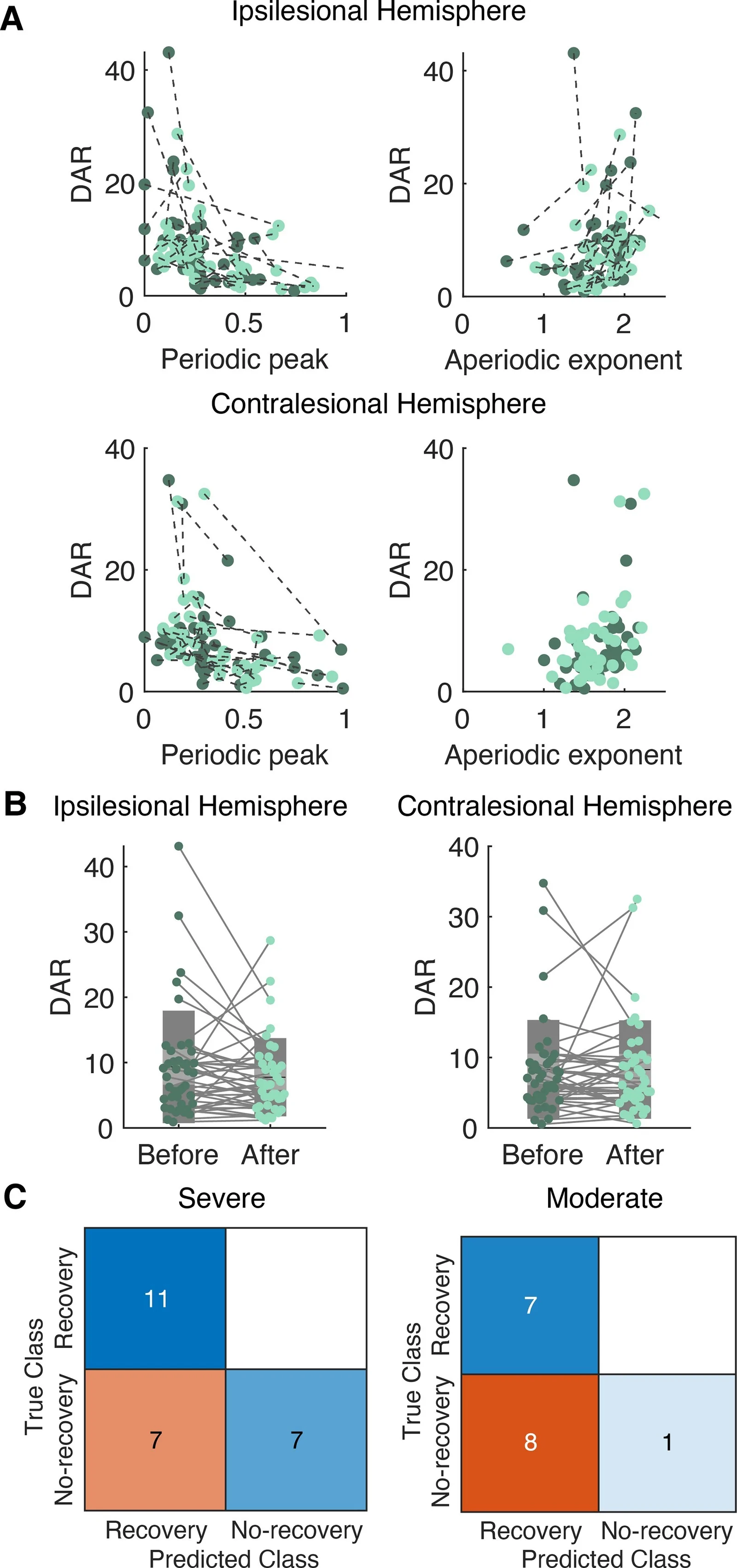
**Relationship between frequency band ratios and spectral parameters.** (**A**) Associations between the delta-to-alpha ratio (DAR) and periodic/aperiodic spectral parameters. Scatter plots show DAR in relation to periodic peak (left) and aperiodic exponent (right) for both the ipsilesional and contralesional hemispheres. (**B**) Changes in DAR before and after the intervention. The left and right panels show data from the ipsilesional and contralesional hemispheres, respectively. (**C**) Confusion matrices for recovery prediction based on a DAR-based model using a leave-one-out cross-validation procedure. Results are shown separately for patients with severe and moderate impairments. Data points: in (**A**), each data point represents one measurement session of one participant (41 participants × 2 sessions = 82 data points), and the line shows the within-participant (repeated measures) fit; in (**B**), each data point represents one participant (*N* = 41) at the pre- or the post-intervention session; in (**C**), each participant contributes one left-out prediction (Severe, *n* = 25; Moderate, *n* = 16). Statistics: (**A**) Repeated measures correlation (df = 40); ipsilesional hemisphere: periodic peak at IAF, *r*_rm_ = −0.375, *P* = 0.014, aperiodic exponent, *r*_rm_ = 0.367, *P* = 0.016; contralesional hemisphere: periodic peak at IAF, *r*_rm_ = −0.52, *P* < 0.001, aperiodic exponent, *r*_rm_ = 0.16, *P* = 0.31. (**B**) Wilcoxon signed-rank test (the Shapiro–Wilk test rejected normality of the paired differences, *P* < 0.01); ipsilesional hemisphere, *W* = 324, *P* = 0.17; contralesional hemisphere, *W* = 406, *P* = 0.76. (**C**) Leave-one-out cross-validation of the DAR-based model; balanced accuracy = 0.75 for the Severe group and 0.56 for the Moderate group.

Then, we tested if the DAR changed after the intervention or correlated with the clinical scores (Supplementary Fig. 6). However, we found neither a significant change nor a within-participant correlation with FMA-UE scores (change: Wilcoxon signed-rank test, ipsilesional hemisphere, *W* = 324, *P* = 0.17; contralesional hemisphere, *W* = 406, *P* = 0.76; the Shapiro–Wilk test rejected normality of the paired differences, *P* < 0.01; association with FMA-UE: repeated measures correlation, ipsilesional hemisphere, *r*_rm_ = −0.12, *P* = 0.43; contralesional hemisphere, *r*_rm_ = −0.01, *P* = 0.93, df = 40). Moreover, the gain prediction was less accurate than the models considering periodic and aperiodic EEG components (Fig. 4B, balanced accuracy for severe patients: 0.75, for moderate patients: 0.56). Combined, the DAR at the chronic stage was a compromised neurophysiological biomarker of functional motor recovery.

## Discussion

In this study, we sought neurophysiological predictors of motor recovery capacity in chronic stroke. Prior EEG-based prognostic work has focused mainly on the acute and subacute phases, where band-power metrics such as DAR correlate with recovery. By contrast, evidence in the chronic stage has been scarce, although technology-driven interventions show that meaningful improvements remain possible.^27^ Our results extend this evidence by demonstrating that EEG-derived characteristics of motor cortical areas were significantly associated with FMA gain, supporting their potential as candidate biomarkers of recovery in chronic stroke.

The significant post-intervention increases in both the IAF peak and SMR proportion in resting EEG suggest that intensive rehabilitation promotes the reorganization of motor cortical circuitry in the ipsilesional hemisphere.^18^ Such use-dependent neuroplastic changes, tightly coupled with functional motor recovery, have been consistently reported in both animal models^28^ and human studies.^29^ In particular, the corticothalamic loop generating alpha oscillations plays a critical role in motor initiation, control, and sensory processing.^30^ The potentiation of alpha oscillations in the chronic stage, therefore, indicates that functional recovery is mediated by strengthened interregional interactions across cortico-subcortical and cortico-cortical networks.

We further demonstrated that the interaction between the aperiodic slope and the periodic peak significantly predicted motor recovery, as indexed by FMA gain. In contrast, DAR, a widely used biomarker in the acute to subacute phase,^11,12^ did not predict recovery in the chronic stage. We interpret this dissociation as evidence that these metrics capture distinct aspects of neural function: while band-ratio measures such as DAR conflate periodic and aperiodic activity, thereby obscuring their differential contributions, the decomposed metrics provide more specific physiological insights related to the sensorimotor system. In particular, increases in low-frequency power (e.g. delta band) are often linked to reduced cognitive capacity or impaired consciousness,^15,16^ which may explain why DAR is predictive in the subacute phase, when fluctuations in consciousness are common, but loses relevance once patients reach a stable chronic state. By contrast, the broadband power reflected in the aperiodic exponent is thought to be related to the local excitatory-inhibitory balance, which is modulated by intrinsic synaptic properties. Given that a larger aperiodic exponent is related to inhibitory-dominant local activity, the interaction between aperiodic and periodic components would reflect motor cortical activity more directly, making their synergistic interplay a mechanistically meaningful marker of recovery potential in chronic stroke.

Despite these promising findings, several limitations should be acknowledged. The sample size was relatively small, particularly after excluding participants due to EEG signal quality, which may limit generalizability. Moreover, the study focused on a short intervention period and resting-state EEG, without integrating structural or functional imaging data. The disparity in balanced accuracy between the severe group (88%) and the Moderate group (64%) can be attributed to several factors, including the smaller sample size of the Moderate group (*n* = 16 versus *n* = 25) reducing model stability, greater variability in FMA gains relative to the group mean. Regarding the prediction model, stepwise regression with a small sample carries a heightened risk of overfitting; although feature selection was reasonably stable across LOOCV folds, these results require external validation in larger cohorts. Supplementary analyses using penalized regression yielded comparable results for the severe group and similarly inconclusive results for the Moderate subgroup. In our cohort, three participants in the moderate group had high baseline FMA-UE scores (56, 58, and 60 out of 66), suggesting that FMA-UE may underestimate functional gains in moderate patients due to ceiling effects; supplementary outcome measures (e.g. Action Research Arm Test) could provide more sensitive assessment in this subgroup.

Our study employed a specific intervention protocol (OT combined with EMG-triggered FES), and it remains to be determined whether the identified EEG biomarkers would generalize to other intensive rehabilitation approaches. However, the EEG features used for prediction were derived from pre-intervention resting-state recordings and thus reflect intrinsic cortical states rather than intervention-specific effects, suggesting that the predictive markers may capture a general capacity for neuroplasticity.

Lesion volume was not collected in the current study as the clinical MRI protocols at our institution did not include standardized volumetric assessment. No formal cognitive evaluation was performed, which we acknowledge as a further limitation, given emerging evidence that cognitive status influences stroke recovery. Our cohort included 16 ischaemic and 25 haemorrhagic stroke patients, but a formal subgroup analysis stratified by stroke type was not performed due to insufficient power in each subgroup; future studies with larger samples should examine whether stroke type modulates the relationship between EEG features and recovery outcomes.

*In the Severe group* duration, while shorter than the recommended 100–120 s for optimal PSD stability, yielded statistically significant and physiologically plausible results; nevertheless, future studies should verify these findings with longer recordings. The stability and specificity of SSD with only three electrodes is inherently lower than with high-density arrays, and future studies should validate these findings with denser montages. Future studies with larger, multicentre cohorts and multimodal approaches are warranted to validate and extend these results. Nevertheless, the present findings highlight the clinical potential of EEG spectral decomposition as a low-cost, non-invasive biomarker to guide personalized rehabilitation strategies and predict recovery trajectories in patients with chronic stroke.

## Supplementary Material

fcag400_Supplementary_Data

## Data Availability

The data that support the findings of this study are available on request from the corresponding author. The data are not publicly available due to their containing information that could compromise the privacy of research participants. The code used for the formal analyses reported in this manuscript is available at https://github.com/Junichi-Ushiba-Laboratory/stroke-analysis.
