# Supplementary material for: Synergistic contributions of periodic and aperiodic cortical activity to motor recovery prediction after stroke
Source: Brain Commun. 2026 Oct 21;8(6):fcag400. doi: 10.1093/braincomms/fcag400 (PMC13729125; doi:10.1093/braincomms/fcag400)
Supplement: Supplementary file 1 [file fcag400_supplementary_data.pdf]

Supplementary Material

"Synergistic contributions of periodic and aperiodic cortical activity to motor recovery prediction after stroke"

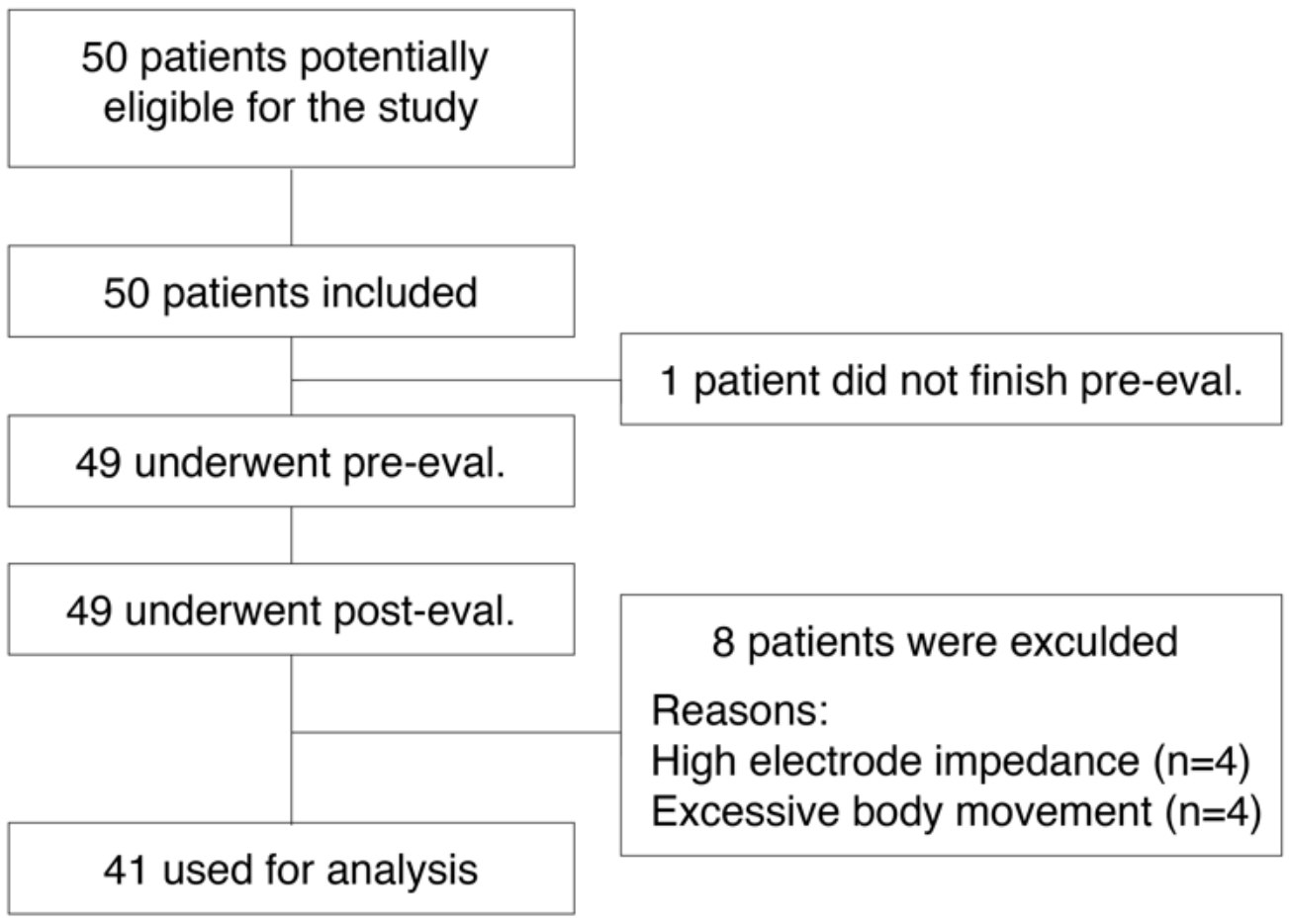

Supplementary Figure 1. Patient flow diagram.

A total of 50 patients were potentially eligible for the study. One patient was excluded because of inability to complete the pre-evaluation. The remaining 49 participants underwent both pre- and post-intervention evaluations. Of these, 8 were further excluded due to insufficient EEG signal quality: high electrode impedance (n = 4) and excessive body movement (n = 4). The final analysis included 41 participants.

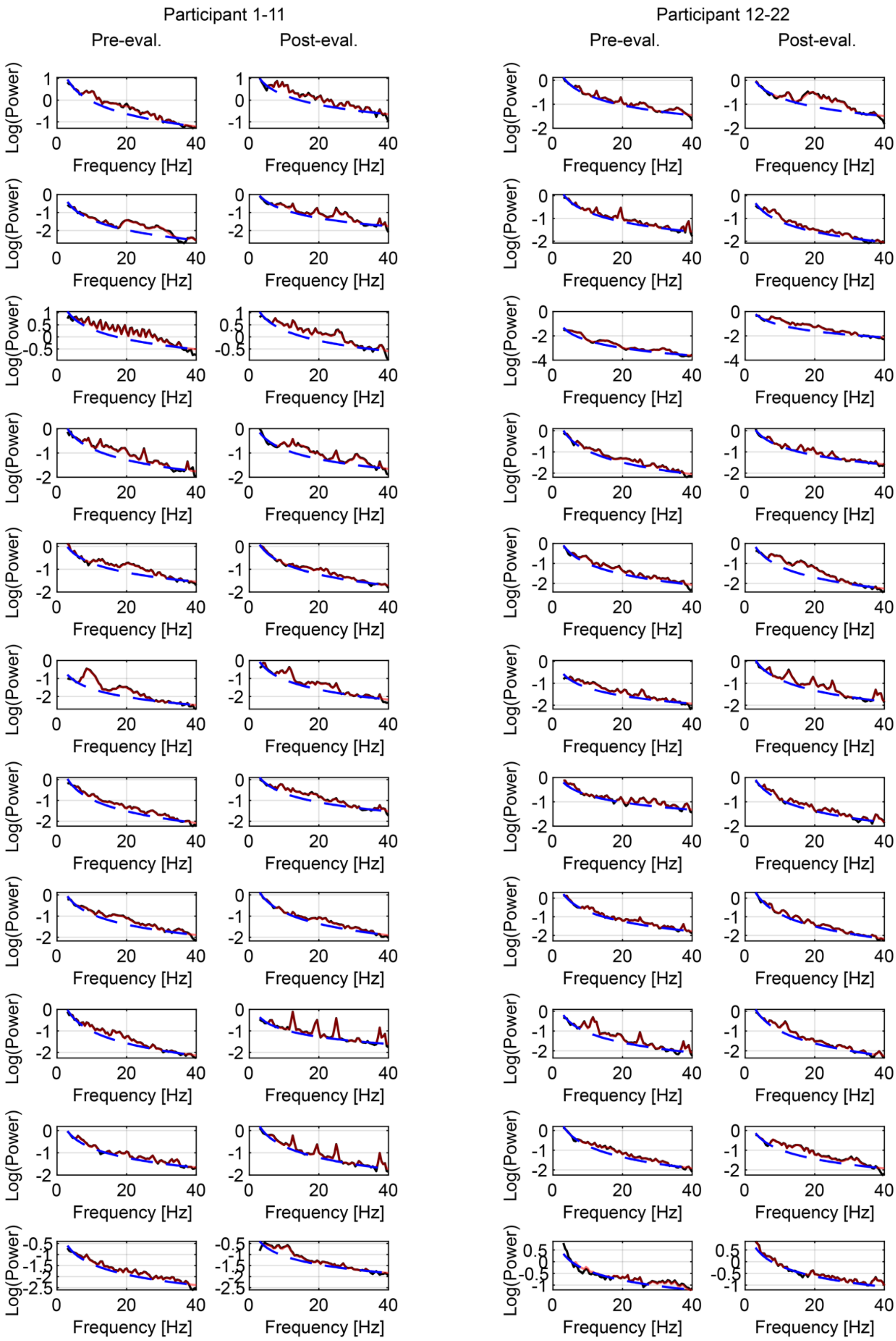

Supplementary Figure 2. Power spectrum data from participant 1-22.

The black data represents raw power spectrum and blue and red lines indicate results of aperiodic and full model fitting derived by *specparam* algorithm.

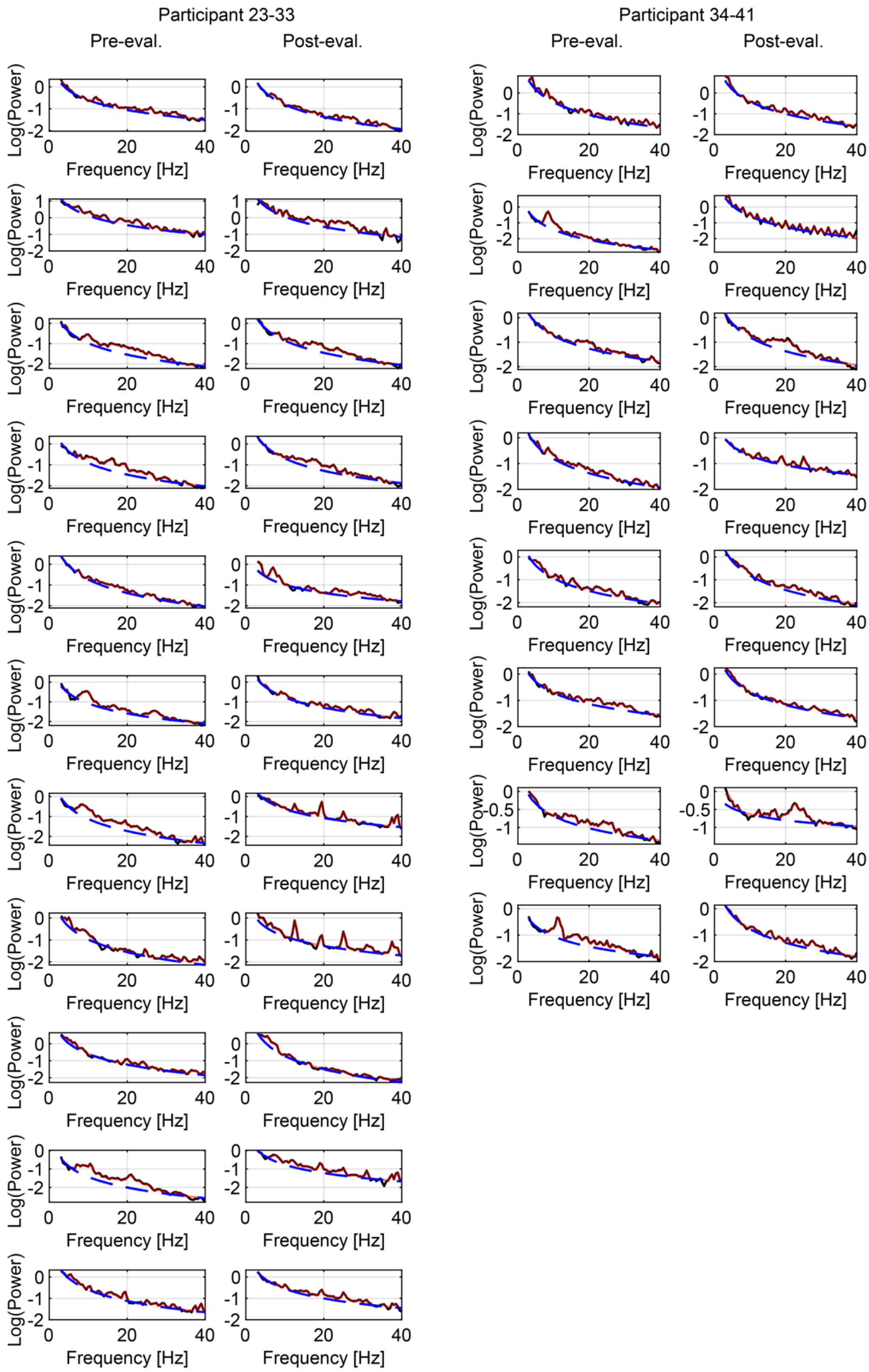

Supplementary Figure 3. Power spectrum data from participant 23-41.

The black data represents raw power spectrum and blue and red lines indicate results of aperiodic and full model fitting derived by *specparam* algorithm.

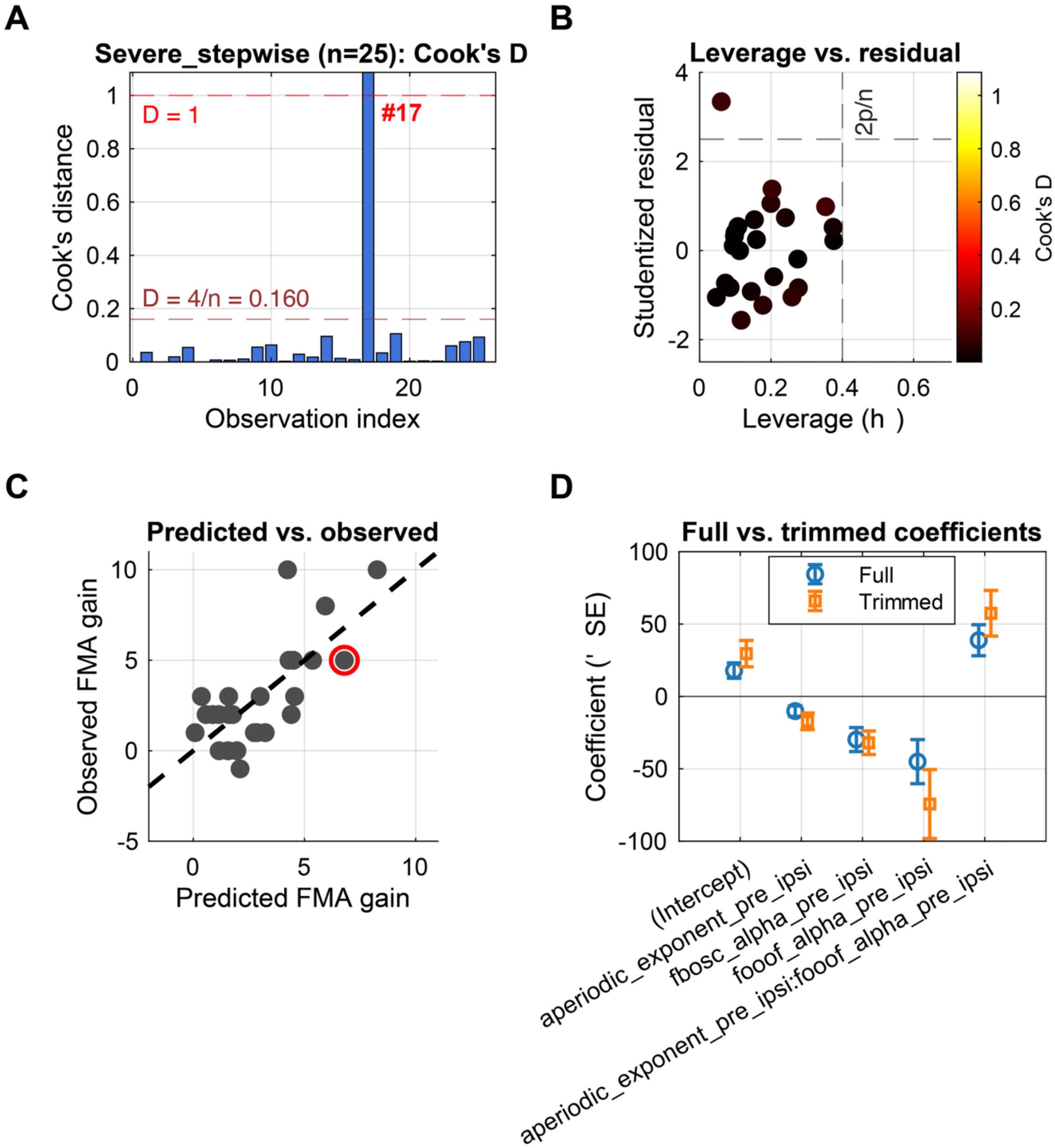

Supplementary Figure 4. Robustness diagnostics for the Severe-group prediction model. The stepwise-selected linear regression model was subjected to influence diagnostics. **(A)** Cook's distance for each observation. The dashed red line denotes the strict threshold $D = 1$ and the dashed brown line denotes the sample-size–adjusted threshold $D = 4/n = 0.160$. Only one observation (#17; FMA gain = 5) exceeded both thresholds (Cook's $D = 1.09$). (B) Leverage (h_{ii}) versus studentized residual, color-coded by Cook's distance. The vertical dashed line marks the leverage threshold $2p/n = 0.400$; no observation simultaneously exceeded the leverage and residual thresholds. (C) Predicted versus observed FMA gain from the full model. The influential observation (#17, red circle) was a mid-range data point and did not drive the

regression fit upward. The dashed line is the identity line. (D) Regression coefficients (estimate ± SE) from the full-sample model (blue circles, n = 25) and from the trimmed model excluding observation #17 (orange squares, n = 24). All coefficients retained their sign, and the periodic × aperiodic interaction term was strengthened after exclusion (β = 38.8 → 57.5). Overall model fit improved upon exclusion (R^2 = 0.532 → 0.576; p = 0.0032 → 0.0019), indicating that the Severe-group prognostic associations are not driven by a small number of influential observations.

Data points: in (A), each bar represents one participant, and in (B) and (C), each data point represents one participant (Severe group, n = 25); in (D), each symbol is a regression coefficient (± standard error) of the full model (n = 25) or the trimmed model (n = 24), estimated from one observation per participant. Predictor labels in (D): fooof_alpha_pre_ipsi, ipsilesional periodic peak at IAF; aperiodic_exponent_pre_ipsi, ipsilesional aperiodic exponent; fbosc_alpha_pre_ipsi, ipsilesional SMR proportion; aperiodic_exponent_pre_ipsi:fooof_alpha_pre_ipsi, periodic × aperiodic interaction. Statistics: (C) and (D) Multiple linear regression (ordinary least squares) including the terms selected by the stepwise procedure in the full sample; the overall model was tested with an F-test and each coefficient with a two-sided t-test. (C) shows in-sample fitted values of the full model (out-of-sample performance is reported in Figure 3B). Full model (n = 25): R^2 = 0.532, $F(4,20)$ = 5.67, p = 0.0032; ipsilesional periodic peak at IAF, β = −45.0, $t(20)$ = −2.96, p = 0.0078; ipsilesional aperiodic exponent, β = −10.1, $t(20)$ = −2.80, p = 0.011; ipsilesional SMR proportion, β = −29.8, $t(20)$ = −3.59, p = 0.0018; periodic × aperiodic interaction, β = 38.8, $t(20)$ = 3.63, p = 0.0017. Trimmed model excluding observation #17 (n = 24): R^2 = 0.576, $F(4,19)$ = 6.46, p = 0.0019; ipsilesional periodic peak at IAF, β = −74.3, $t(19)$ = −3.12, p = 0.0057; ipsilesional aperiodic exponent, β = −17.2, $t(19)$ = −3.01, p = 0.0072; ipsilesional SMR proportion, β = −32.0, $t(19)$ = −3.94, p = 0.00088; periodic × aperiodic interaction, β = 57.5, $t(19)$ = 3.64, p = 0.0017.

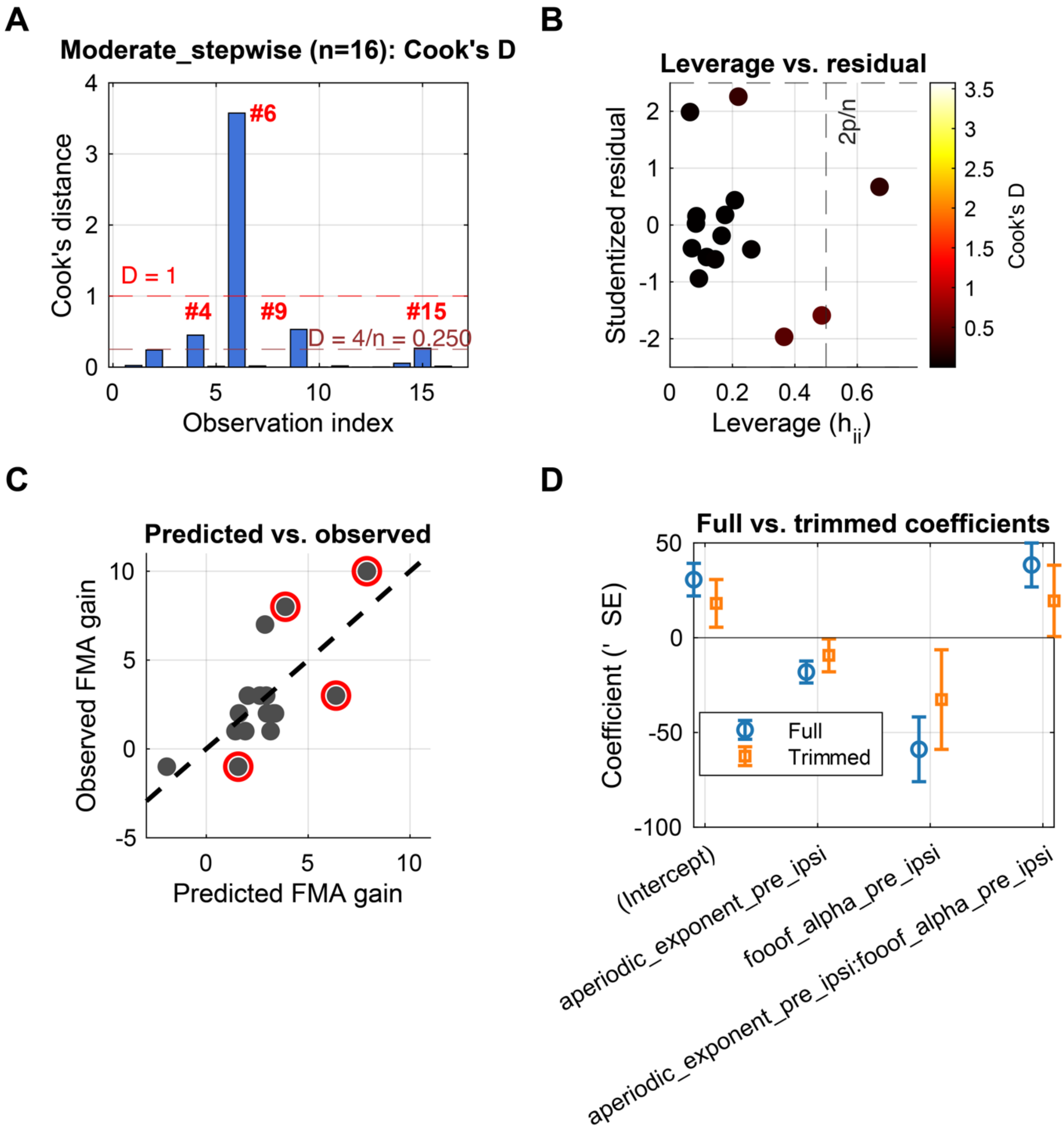

Supplementary Figure 5. Robustness diagnostics for the Moderate-group prediction model. The stepwise-selected linear regression model was subjected to the same influence diagnostics. (A) Cook's distance per observation; dashed red line: $D = 1$, dashed brown line: $D = 4/n = 0.250$. Four observations (#4, #6, #9, #15) exceeded the 4/n threshold, and observation #6 (FMA gain = 10) markedly exceeded the strict threshold ($D = 3.57$). (B) Leverage versus studentized residual, color-coded by Cook's distance. The vertical dashed line indicates the leverage threshold 2p/n = 0.500. Two observations exceeded the leverage threshold, and the most influential point (#6) showed both high leverage ($h_{ii} = 0.79$) and a large positive residual. (C) Predicted versus observed FMA gain. Observations flagged by the 4/n criterion (red circles) included the two largest-recovery cases (FMA gain = 10 and 8) and one case with FMA gain

= 3, together with one non-recovering case (FMA gain = −1). The dashed line is the identity line. (D) Regression coefficients (estimate ± SE) from the full model (blue circles, n = 16) and the trimmed model excluding the four influential observations (orange squares, n = 12). Excluding these observations substantially attenuated all coefficients, and the overall model was no longer statistically significant (R^2 = 0.502 → 0.347; p = 0.034 → 0.308). These results indicate that the Moderate-group prediction model is sensitive to a small number of influential observations consistent with the limited sample size, and the findings should therefore be interpreted as exploratory.

Data points: in (A), each bar represents one participant, and in (B) and (C), each data point represents one participant (Moderate group, n = 16); in (D), each symbol is a regression coefficient (± standard error) of the full model (n = 16) or the trimmed model (n = 12), estimated from one observation per participant. Predictor labels in (D) are as defined in Supplementary Figure 4. Statistics: (C) and (D) Multiple linear regression (ordinary least squares) including the terms selected by the stepwise procedure in the full sample; the overall model was tested with an F-test and each coefficient with a two-sided t-test. (C) shows in-sample fitted values of the full model (out-of-sample performance is reported in Figure 3B). Full model (n = 16): $R^2 = 0.502$, $F(3,12) = 4.02$, $p = 0.034$; ipsilesional periodic peak at IAF, $\beta = -58.9$, $t(12) = -3.45$, $p = 0.0048$; ipsilesional aperiodic exponent, $\beta = -18.1$, $t(12) = -3.13$, $p = 0.0088$; periodic × aperiodic interaction, $\beta = 38.4$, $t(12) = 3.31$, $p = 0.0062$. Trimmed model excluding observations #4, #6, #9 and #15 (n = 12): $R^2 = 0.347$, $F(3,8) = 1.42$, $p = 0.308$; ipsilesional periodic peak at IAF, $\beta = -32.6$, $t(8) = -1.24$, $p = 0.25$; ipsilesional aperiodic exponent, $\beta = -9.3$, $t(8) = -1.07$, $p = 0.31$; periodic × aperiodic interaction, $\beta = 19.4$, $t(8) = 1.03$, $p = 0.33$.

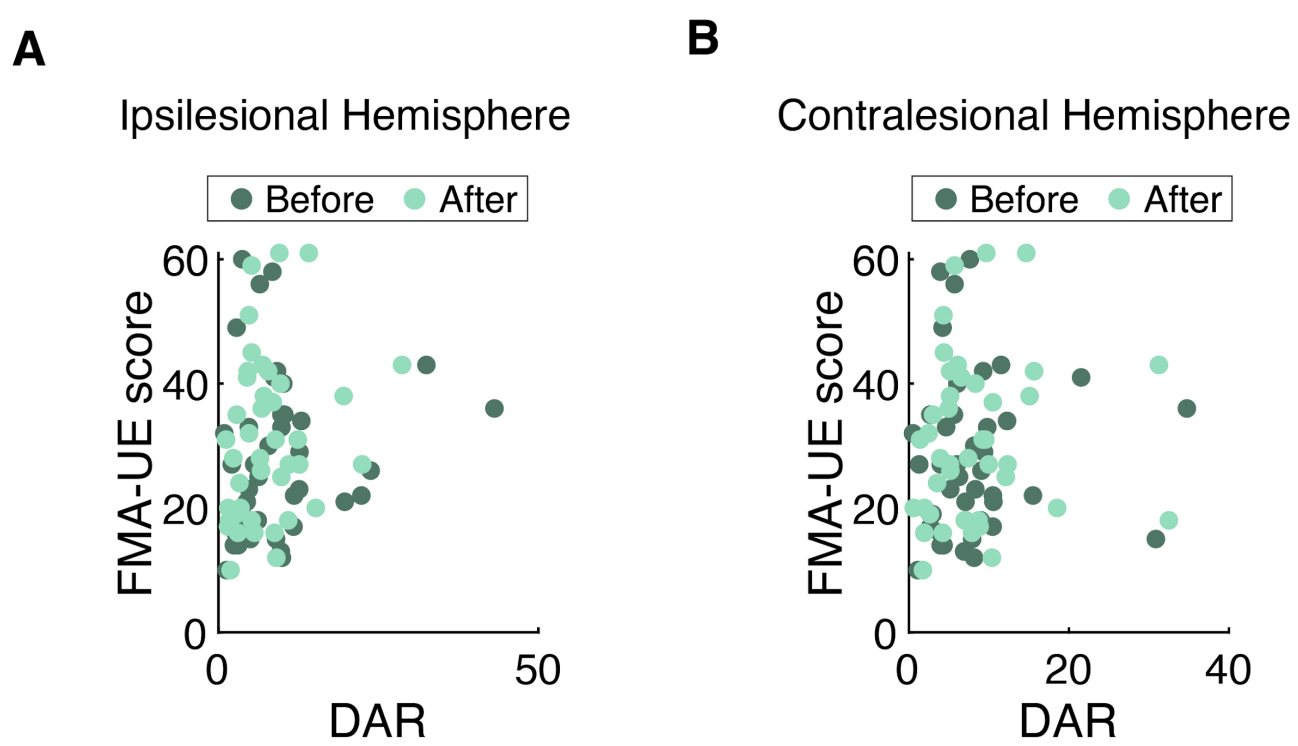

Supplementary Figure 6. Relationship between FMA-UE scores and delta-alpha ratio. (A) Scatter plots showing the relationship between FMA-UE scores and delta-alpha ratio from the ipsilesional hemisphere. (B) Scatter plots showing the relationship between FMA-UE scores and delta-alpha ratio from the contralesional hemisphere.

Data points: each data point represents one measurement session of one participant (41 participants × 2 sessions = 82 data points; dark green, before the intervention; light green, after the intervention). Statistics: Repeated measures correlation (df = 40); (A) ipsilesional hemisphere, $r_{rm} = -0.12$, $p = 0.43$; (B) contralesional hemisphere, $r_{rm} = -0.01$, $p = 0.93$.
